# 

**Published:** 1843-04

**Authors:** 


### CONTENTS OF No. XXX.

OF THE

### British and Foreign Medical Review.

APRIL, 1843.

---

### Part First.—Analytical and Critical Reviews.

PAGE

Art. I.—1. The Effects of Arts, Trades, and Professions, and of Civic States and Habits of Living, on Health and Longevity, with suggestions for the removal of many of the agents which produce disease, and shorten the duration of life. By C. Turner Thackrah, Esq. . . . . 285

2. Report from the Committee on the Bill to regulate the Labour of Children in the Mills and Factories of the United Kingdom; with the Minutes of Evidence, Appendix, and Index. Ordered by the House of Commons to be printed, 1832 . . . . . . . ib.

3. The Philosophy of Manufactures: or an Exposition of the Scientific, Moral, and Commercial Economy of Great Britain. By Andrew Ure, m.d. f.r.s. ib.

4. Report from the Select Committee on the Health of Towns; together with the Minutes of Evidence taken before them, and an Appendix and Index. Ordered by the House of Commons to be printed, 1840 . . ib.

5. Tableau de l'Etat Physique et Moral des Ouvriers employés dans les Manufactures de Coton, de Laine, et de Soie. Ouvrage entrepris par ordre de l'Académie des Sciences Morales et Politiques. Par M. le Dr. Villermé . ib.
Sketch of the Physical and Moral Condition of the Workpeople employed in the Manufactures of Cotton, Wool, and Silk. A work undertaken by order of the Academy of Moral and Political Sciences. By Dr. Villermé.

6. Third Annual Report of the Registrar-General of Births, Deaths, and Marriages in England, with Appendices. Presented to both Houses of Parliament by Command of Her Majesty . . . . . ib.

7. Notes of a Tour in the Manufacturing Districts of Lancashire; in a Series of Letters to His Grace the Archbishop of Dublin. By W. Cooke Taylor, ll.d. &c. &c. . . . . . . ib.

8. Report to Her Majesty's principal Secretary of State for the Home Department, from the Poor Law Commissioners, on an Inquiry into the Sanatory Condition of the Labouring Population of Great Britain; with Appendices. Presented to both Houses of Parliament by Command of Her Majesty. Drawn up by Edwin Chadwick, Esq. . . . . . 286

Art. II.—Dissertatio Obstetrica Inauguralis de Prolapsu Funiculi Umbilicalis. Auctore Joh. Christ. Saxtorph . . . . . 314
Obstetric Inaugural Dissertation on Prolapsus of the Umbilical Cord. By J. C. Saxtorph.

§

PAGE

Art. III.—1. Traité Pratique des Maladies des Enfants, depuis la Naissance jusqu'à la Puberté. Par E. A. J. Berton, d.m. &c. . . . 320
A Practical Treatise on the Diseases of Children, from birth to the period of puberty. By Dr. E. A. J. Berton.
2. Traité pratique des Maladies de l'Enfance. Par F. Barrier, d.m. Tome Premier . . . . . . . ib.
A Practical Treatise on the Diseases of Childhood. By Dr. Barrier. Vol. I.

Art. IV.—1. General Report on the Sanatory Condition of the Labouring Population of Great Britain. By Edwin Chadwick, Esq. . . . 328
2. Local Reports on the Sanatory Condition of the labouring Population of Great Britain, in consequence of an Inquiry directed to be made by the Poor Law Commissioners. Two Vols. Vol. I.—England and Wales. Vol. II.—Scotland. ib.

Art. V.—1. Des Pertes Seminales Involontaires. Par M. Lallemand . 346
On Involuntary Seminal Discharges. By M. Lallemand.
2. Mémoire sur l'Emploi des Caustiques dans quelques Maladies de l'Urètre. Par le Dr. Civiale . . . . . . . ib.
Memoir on the Employment of Caustics in some diseases of the Urethra. By Dr. Civiale.
3. De Vesiculis Seminalibus Dissertatio. Auctore F. C. Faye, m.d. . ib.
A Dissertation on the Seminal Vesicles. By Dr. Faye.

Art. VI.—Elements of General Pathology. By the late John Fletcher, m.d. Edited by John J. Drysdale, m.d. and John R. Russell, m.d. . . 367

Art. VII.—1. A Practical and Theoretical Treatise on the Diagnosis, Pathology, and Treatment of Diseases of the Skin; arranged according to a natural System of Classification, and preceded by an Outline of the Anatomy and Physiology of the Skin. By Erasmus Wilson . . . 372
2. Manual of Diseases of the Skin. From the French of MM. Cazenave and Schedel. With Notes and Additions by Thomas H. Burgess, m.d. . ib.
3. Nouvelle Dermatologie, ou Précis théorique et pratique sur les Maladies de la Peau, fondé sur une nouvelle Classification médicale, avec un Formulaire special et Planches coloriées. Par P. Baumès . . . ib.
New Dermatology; or a Theoretical and Practical Treatise on Diseases of the Skin, founded upon a new medical Classification, with a special Formulary and coloured Plates. By P. Baumes.

Art. VIII.—1. Heelkundige Mengelingen. Door J. F. Kerst . . 399
Surgical Miscellanies. By J. F. Kerst.
2. Klinische Bijdragen tot de Theorie en Praktijk der Genees en Heelkunde. Door C. Gobee . . . . . . ib.
Clinical Contributions to the Theory and Practice of Medicine and Surgery. By C. Gobee.

Art. IX.—1. The Simple Treatment of Disease; deduced from the methods of Expectancy and Revulsion. By James M. Gully, m.d. . . 405
2. Beiträge zur wissenschaftlichen Heilkunde. Von Dr. C. A. W. Richter . ib.
Contributions to Scientific Medicine. By Dr. C. A. W. Richter.

Art. X.—The Reciprocal Influence of Body and Mind considered; as it affects the great questions of Education, Phrenology, Materialism, Moral Advancement, and Responsibility,—Man's free agency, the theory of life, the peculiarities of mental property, mental diseases, the agency of mind upon the body, of physical temperament upon the manifestations of mind and upon the expression of religious feelings. By W. Newnham, Esq. m.r.s.l. . . 413

Art. XI.—1. Diss. Med. Inauguralis de Cancro Pulmonum. Auctore E. N. Van Kleffens . . . . . . . 430
Inaugural Dissertation on Cancer of the Lungs. By E. N. Van Kleffens.
2. Cases of Malignant Disease of the Lung. By H. M. Hughes, m.d. (Guy's Hospital Reports, No. XIII.—October, 1841) . . . ib.

PAGE

3. Clinical Lecture on Cancer of the Lung, delivered at University College Hospital. By Dr. Taylor. (Lancet, March 26, 1842) . . . 430

4. Researches on the Pathology and Diagnosis of Cancers of the Lung and Mediastinum. By Willam Stokes, M.D. (Dublin Journal of Medical Science, May, 1842) . . . . . . . ib.

5. The Physical Diagnosis of Diseases of the Lungs, (Chapter relating to Cancer of the Lungs.) By W. H. Walshe, M.D. . . . . ib.

Art. XII.—Theophili Protospatharii de corporis humani fabrica, Libri V. Edidit Guilielmus Alexander Greenhill, M.D. . . . 439
The Five Books of Theophilus Protospatharius on the Construction of the Human Body. Edited by William Greenhill, M.D.

Art. XIII.—An Inquiry into the Nature and Causes of Epilepsy; with the Function of the Spleen and the use of the Thyroid Body, &c. &c. By John Jackson 455

Art. XIV.—Commentaries on some Doctrines of a dangerous tendency in Medicine, and on the general principles of safe Practice. By Sir Alexander Crichton, M.D. F.R.S. L.S. & G.S. &c. &c. . . . . 459

Art. XV.—Traité des Maladies des Reins, et des Altérations de la Sécrétion Urinaire, &c. Avec un Atlas in Folio. Par P. Rayer. Tome III. . 470
Treatise on the Diseases of the Kidneys and the Morbid States of the Urinary Secretion. With Folio Plates. By P. Rayer. Vol. III.

Art. XVI.—Treatment of the Diseases of the Eye by means of Prussic Acid Vapour, and other Medicinal Agents. By A. Turnbull, M.D. . 494

Art. XVII.—On Gravel, Calculus, and Gout; chiefly an application of Professor Liebig's Physiology to the Prevention and Cure of these Diseases. By Henry Bence Jones, M.A. . . . . . 500

Art. XVIII.—1. Report on the Effect of Interment of Bodies on the Health of Towns. Ordered by the House of Commons to be printed, 14th June, 1842. 513

2. Health of Towns. A Bill for the Improvement of Health in Towns, by removing the Interment of the Dead from their Precincts. Prepared and brought in by Mr. Mackinnon, Mr. Cowper, and Mr. Beckett, 5th Aug. 1842 . . . . . . . ib.

3. Ueber den Einflusz der Verwesungsdünste auf die menschliche Gesundheit, und über die Begrabnissplätze in Medizinisch-politzeilicher Beziehung. Von Dr. V. A. Riecke . . . . . . ib.
On the Influence of Putrefactive Emanations on the Health of Man, and on Burial Grounds in their relation to Medical Police. By Dr. V. A. Riecke.

Art. XIX. — A Treatise on Protracted Indigestion, and its Consequences, Addressed by the Author, on his retirement from the medical profession, both to the members of that profession and to the well-educated public, particularly parents. By A. P. W. Philip, M.D. F.R.S. . . . 524

Art. XX.—Practical Observations on Midwifery, with Cases in Illustration. By John Ramsbotham, &c. &c. . . . . . 527

Art. XXI.—1. Remarks on Medical Reform, in a Letter addressed to the Right Hon. Sir James Graham, Bart. &c. By Sir James Clark, M.D. F.R.S. &c. 529

2. Remarks on Medical Reform, in a Second Letter addressed to the Right Hon. Sir James Graham, Bart. By Sir James Clark, Bart. M.D. F.R.S. &c. . ib.

Art. XXII.—Elements of Chemical Analysis, Inorganic and Organic. By Edward A. Parnell . . . . . . . 531

Art. XXIII.—1. Outlines of Pathology and Practice of Medicine. By W. P. Alison, M.D. F.R.S.E. Parts I. II.—Preliminary Observations: Inflammatory and Febrile Diseases . . . . . . 533

2. Methodus Medendi; or the Description and Treatment of the principal Diseases incident to the Human Frame. By H. M'Cormac, M.D. . . ib.

### PART SECOND.—Bibliographical Notices.

PAGE

ART. I.—The Connexion between Physiology and Intellectual Philosophy. By the Rev. JOHN BARLOW, M.A. F.R.S. F.L.S. &c. &c. . . . 535

ART. II.—The Hunterian Oration, delivered at the Royal College of Surgeons in London on the 14th February, 1843. By J. M. ARNOTT . . 536

ART. III.—The Physiological Anatomy and Physiology of Man. By ROBERT B. TODD, M.D. F.R.S. and WILLIAM BOWMAN, F.R.S. . . . 537

ART. IV.—On the Chemical Discrimination of Vesical Calculi. By E. SCHARLING. Translated from the Latin, with an Appendix, by S. E. HOSKINS, M.D. . ib.

ART. V.—Chemical Manipulation; being Instructions to Students in Chemistry on the Methods of performing Experiments of Demonstration or Research, with Accuracy and Success. By MICHAEL FARADAY, D.C.L. F.R.S. &c. &c. 538

ART. VI.—The Causes, Nature, Diagnosis, and Treatment of Acute Hydrocephalus. By J. R. BENNETT, M.D. . . . . . 539

ART. VII.—1. A Practical Treatise on Pulmonary Consumption, its Pathology, Diagnosis, and Treatment, &c. By FRANCIS COOK, M.D. M.R.C.S.E. . 540

2. An Exposition of the Pathology and Treatment of Tubercular Phthisis. By SAMUEL FLOOD, M.R.C.S. . . . . . ib.

ART. VIII.—A New Theory and Treatment of Disease, founded on Natural Principles. By JOHN TINNION, M.D. AYR . . . . ib.

ART. IX.—Elements of Electro-Metallurgy. By ALFRED SMEE, F.R.S. . . 541

ART. X.—Thermal Comfort; or Popular Hints for Preservation against Colds, Coughs, and Consumption. By SIR GEORGE LEFEVRE, M.D. &c. . ib.

ART. XI.—Report on the Chief Results obtained by the use of the Microscope in the Study of Human Anatomy and Physiology. By JAMES PAGET . 542

ART. XII.—Interment and Disinterment; or a Further Exposition of the Practices pursued in the Metropolitan Places of Sepulture, as affecting the Health of the Living. By S. A. WALKER . . , . . ib.

### PART THIRD.—Original Reports and Memoirs.

1. Clinical and Pathological Report on the Pneumonia of Children, as it prevails among the Poor in London. With a Tabular View of Cases of Lobar, Lobular, and Vesicular Pneumonia. By CHARLES WEST, M.D. . . 543

### PART FOURTH.—Medical Intelligence.

1. The Sydenham Society, with a Prospectus, and a List of the Provisional Officers . . . . . . . . 573

2. List of Original Papers in the British Medical Journals for the quarter ending the 20th March, 1843 . . . . . . . 575

3. BOOKS RECEIVED FOR REVIEW . . . . . . . 577

TITLE, INDEX, &c.

